# Long term treatment with omalizumab in adolescent with refractory solar urticaria

**DOI:** 10.1186/s13052-021-01151-z

**Published:** 2021-09-28

**Authors:** Mauro Iannelli, Stefano Passanisi, Giuseppe Crisafulli, Stefania Arasi, Lucia Caminiti, Giuseppina Zirilli, Giovanni B. Pajno

**Affiliations:** 1 Department of Human Pathology in Adult and Developmental Age “Gaetano Barresi”, Allergy Unit, University of Messina, Via Consolare Valeria 1, 98124 Messina, ME Italy; 2 Predictive and Preventive Medicine Research Unit, Multifactorial and Systemic Disease Research Area, Pediatric Allergology Unit, Bambino Gesù Children’s Research Hospital (IRCCS), Rome, Italy

**Keywords:** Anti-IgE, Chronic inducible urticaria, Management, Quality of life

## Abstract

**Background:**

Solar urticaria represents an uncomfortable form of chronic inducible urticaria. First and second-line treatments are ineffective in some patients, leading to an impairment in their quality of life. Omalizumab represents a safe therapeutic option in case of refractory solar urticaria.

**Case presentation:**

We update a case of a 21-year-old Caucasian girl affected by solar urticaria from the age of 14. Poor disease control was achieved with standard or high-dose of H_1_-antihistamines. Several omalizumab courses, including a 1-year-long course, were practiced resulting in clinical remission and significant improvement in patient’s quality of life.

**Conclusion:**

Our experience confirms the effectiveness and safety of omalizumab for the management of refractory solar urticaria. Future studies are awaited in order to monitor long term effects and chronic doses of this treatment, particularly in patients who need concomitant therapy with antihistamines.

## Background

Solar urticaria (SU) represents a quite rare but uncomfortable form of chronic inducible urticaria. It is caused by sun-exposure and ultraviolet (UV) irradiation, UVB and more frequently UVA. Skin manifestations usually appear after few minutes of sunlight exposure. Because of its rarity, there are few data on the prevalence and incidence. SU usually manifests itself in the fourth decade of life and it occurs more commonly in women than men, regardless skin phototype and ethnicity [1]. First line treatment is based on second-generation H_1_-antihistamines, which can be increased up 2–4 fold the standard dosage to achieve clinical remission. However, standard therapy with antihistamines is ineffective in some patients; therefore, these patients require second and third-line therapeutic approaches (e.g. antileukotrienes, cyclosporine-A, biologics) along with absolute avoidance of sun exposure. The disease-related limitation of normal daily activity frequently leads to an impairment in quality of life of patients. Omalizumab, a recombinant humanized anti-IgE monoclonal antibody approved for the management of chronic spontaneous urticaria and severe asthma, has been recently demonstrated as a safe and effective therapeutic option in patients with inadequate response to standard therapies [2–5]. In Italy, omalizumab is approved for use in chronic spontaneous urticarial, but it is considered off-label for the management of chronic inducible urticaria, including SU [6].

## Case presentation

**Fig. 1:**
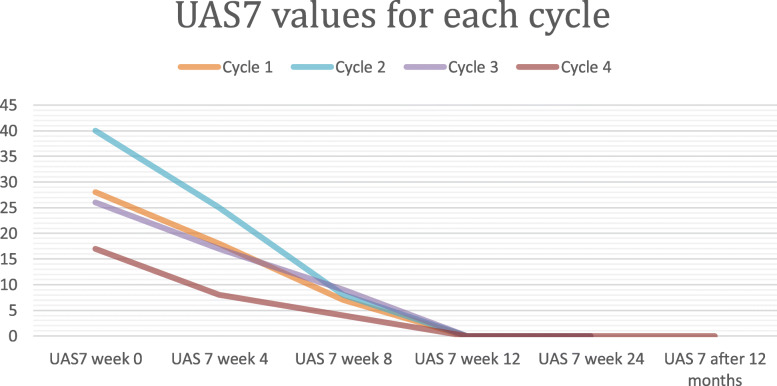
Evaluation of UAS7 values at each omalizumab course

We update a previously described case of a 21-year-old Caucasian girl suffering from SU from the age of 14 [7]. She was firstly treated with three H_1_-antihistamines at standard dose for a total of 8 months (cetirizine 10 mg/day, desloratadine 5 mg/day and hydroxyzine 25 mg/day) with unsatisfactory response to therapy. Treatment with H_1_-antihistamine at double dose (cetirizine 20 mg/day) associated to with H_2_-antihistamine at standard dose (ranitidine 150 mg/day) and antileukotriene (montelukast 10 mg/day) was also ineffective. During the first 2 years of disease, several 5-day courses of oral prednisone (25 mg daily) were prescribed as add-on therapy. However, the patient had a poor disease control. At the age of 16 she underwent a 9-month course of experimental therapy with omalizumab subcutaneously, with a starting dose of 375 mg every 2 weeks for 6 months which was progressively decreased to a monthly 150 mg maintenance dose up to suspension. Therapeutic response of the patient had been promptly obtained after the first administration and clinical improvement persisted when maintenance dose had been practiced. Clinical remission was confirmed by the negative results of photo-test for UVA and UVB which was performed at the end of omalizumab course and after 4 months from discontinuation of therapy (Fig. 1).

During the subsequent 4 years, the patient underwent a total of 4 courses of subcutaneous omalizumab due to recurrent relapses of SU after sun exposure. Laboratory tests, including serum IgE levels, eosinophil count, inflammation markers and antinuclear antibodies, were performed at the beginning of each course. Disease activity was monitored by the Urticaria Activity Score over 7 days (UAS7). The first three therapeutic courses were practiced at the dose of 300 mg monthly for a total of 6 months. During treatment cutaneous symptoms faded to a complete remission, as demonstrated by the reduction of UAS7 score values (Fig. 1). Short treatment with second-generation antihistamines at standard dose was occasionally required during omalizumab course due to the appearance of mild cutaneous manifestations (rupatadin 10 mg/day or bilastine 20 mg/day) (Table 1).

**Table 1:** Occasional relapses of SU during the 4-year period of follow-up and required treatment

	***Cycle 1***	***Cycle 2***	***Cycle 3***	***Cycle 4***
***Number of flares***	0	2	0	2
***Clinical manifestations***	–	Erythema/Mild itch	–	Erythema, hives, angioedema/mild itch
***Treatment description***	–	Rupatadine (10 mg/day for 14 days)	–	Rupatadine (10 mg/day for 7 days) Bilastine (20 mg/day for 7 days)
***Remission interval after last administration (months)***	4	3	1	4

Symptoms appeared within just 1 month from the last administration of the third omalizumab therapeutic course. Thus, we decided to prescribe a prolonged course of treatment. The girl underwent omalizumab therapy at the previous tested effective dose of 300 mg monthly for 1 year continuously. The aim of this long lasting treatment was to achieve a more sustained response and persistent remission, according to scientific evidence [8, 9].

A complete clinical remission reconfirmed to be gained starting from the third dose and was associated with a sustained negativity of UAS7 values. At the 10th month of this treatment, standard dose of anti-histamines (rupatadin 10 mg/day for 7 days and following bilastine 20 mg/day for 7 days) was successfully practiced due to the appearance of two small flares resulting in itch and moderate sunlight-induced erythema. Photo-tests performed at the end of therapy resulted negative. No adverse effects were detected during all the omalizumab courses. Dermatology Life Quality Index (DLQI) questionnaire was completed by the patient to assess the impact of SU on quality of life prior to omalizumab treatment, at the end of both the first and last omalizumab courses. DLQI score was 24, 12 and 9, respectively, confirming a significant reduction of the emotional burden of the disease.

## Discussion and conclusion

Omalizumab is a recombinant humanized monoclonal antibody that binds to the CHε3 region of IgE and, thus, avoids IgE from binding to FcεRI and FcεRII receptors on the surface of mast cells and basophils [10]. Omalizumab also prevents IgE from binding to FcεRI on dendritic cells and this could be related to a decreased release of inflammatory mediators with subsequent inhibitory effects on the allergen cascade [11]. Long-term therapy with omalizumab has been approved for the management of moderate to severe asthma and allergic rhinitis. It is also considered effective and safe in treating vernal keratoconjunctivitis and as an add-on maintenance treatment of nasal polyps [12–14]. The 2017 EAACI/GA2LEN/EDF/WAO international guidelines recommend the use of omalizumab as third–line of treatment for the management of CSU in adults and adolescents aged ≥12 years with inadequate response to H_1_-antihistamines [15]. Omalizumab use in patients younger than 12 years is still off-label for CSU despite emerging data suggesting safety and efficacy in this population [16].

Regarding chronic inducible urticaria, including SU, there are sparse reports on the use of omalizumab both in adult and pediatric patients [17, 18]. A recent systematic review, covering a total of 48 patients with antihistamine refractory SU, reported that treatment with omalizumab resulted in clinical improvement for 80% of patients, and 50% of them became symptom free. About 20% of patients failed to achieve any clinical response under a monthly dosage of 150–300 mg of omalizumab. Mild adverse events (i.e. gastrointestinal intolerance, periorbital edema, local reaction, and short-term asthenia) were recorded in only 11% of subjects [8]. However, most studies have short follow-up periods which are limited to a few months.

To the best of our knowledge, this is the first report describing a 4-year follow-up on omalizumab use to treat an adolescent patient with refractory SU. Our case confirms the rapid clinical efficacy and safety of 300 mg monthly dose. Noteworthy, a similar experience of long-term therapy with omalizumab plus low dose anti-histamines has been reported in a child with SU who was treated for 36 months resulting in clinical remission [8]. In recent years, research teams marked the importance to identify reliable biomarkers which are able to predict changes in disease activity as treatment response. Among these, baseline IgE levels appear to be the most intriguing factor. Some studies showed that among patients with chronic urticaria, non-responders to omalizumab had significantly lower baseline IgE levels than partial responders and complete responders [19, 20]. More specifically, low baseline serum IgE < 15.2 IU/mL would predict a lower likelihood of response to omalizumab [19]. Our case supports this finding since IgE values varied from 73.7 to 224.5 IU/ml before each therapeutic course, thus defining our patient as a full-responder.

Another relevant aspect that should be taken into account in SU patients is the psychological impact. Quality of life impairment is quite common among adults and children suffering from SU, especially during relapse times [21]. Can et al. have recently demonstrated that omalizumab not only provides symptom control for chronic urticaria but also improves patients’ psychological conditions, thereby making the disease less hard to manage [22]. Likewise, our patient reported a progressive improvement in quality of life as demonstrated by the reduction of DLQI questionnaire scores.

Finally, our experience regarding the last one-year course contributes to support information on efficacy, safety, and tolerability of a prolonged therapeutic regimen and strengthens the awareness that SU cases unresponsive to topical treatment and cyclosporin may need omalizumab [6, 8, 9]. However, unavoidable short exposure to UV, especially in sunny countries, facilitate the occurrence of recurrent relapses of SU after omalizumab discontinuation. In this context, the need of sustained omalizumab treatment at low dosage should be considered in the near future. Therefore, randomized controlled trials with long lasting follow-up are awaited to monitor the chronic doses of omalizumab and schedules for eligible patient suffering from solar urticaria unresponsive to conventional treatment.

## Data Availability

Not applicable.

## Abbreviations

DLQI: Dermatology Life Quality Index
SU: Solar Urticaria
UV: Ultraviolet
UAS: Urticaria Activity Score
